# Quantifying allo-grooming in wild chacma baboons (*Papio ursinus*) using tri-axial acceleration data and machine learning

**DOI:** 10.1098/rsos.221103

**Published:** 2023-04-12

**Authors:** Charlotte Christensen, Anna M. Bracken, M. Justin O'Riain, Gaëlle Fehlmann, Mark Holton, Phillip Hopkins, Andrew J. King, Ines Fürtbauer

**Affiliations:** ^1^ Faculty of Science and Engineering, Swansea University, Swansea SA2 8PP, UK; ^2^ Department of Evolutionary Biology and Environmental Science, University of Zurich, Zurich 8057, Switzerland; ^3^ School of Biodiversity, One Health and Veterinary Medicine, University of Glasgow, Glasgow G12 8QQ, UK; ^4^ Institute for Communities and Wildlife in Africa, Department of Biological Science, University of Cape Town, Rondebosch, 7701, South Africa; ^5^ Max Planck Institute of Animal Behavior, 78315 Radolfzell, Germany

**Keywords:** machine learning, tri-axial accelerometers, random forest models, allo-grooming, activity budgets, primates

## Abstract

Quantification of activity budgets is pivotal for understanding how animals respond to changes in their environment. Social grooming is a key activity that underpins various social processes with consequences for health and fitness. Traditional methods use direct (focal) observations to calculate grooming rates, providing systematic but sparse data. Accelerometers, in contrast, can quantify activity budgets continuously but have not been used to quantify social grooming. We test whether grooming can be accurately identified using machine learning (random forest model) trained on labelled acceleration data from wild chacma baboons (*Papio ursinus*). We successfully identified giving and receiving grooming with high precision (81% and 91%) and recall (87% and 79%). Giving grooming was associated with a distinct rhythmical signal along the surge axis. Receiving grooming had similar acceleration signals to resting, and thus was more difficult to assign. We applied our machine learning model to *n* = 680 collar data days from *n* = 12 baboons and found that grooming rates obtained from accelerometers were significantly and positively correlated with direct observation rates for giving but not receiving grooming. The ability to collect continuous grooming data in wild populations will allow researchers to re-examine and expand upon long-standing questions regarding the formation and function of grooming bonds.

## 1. Introduction

How animals allocate time and energy to different activities has important fitness consequences [1]. The observed activity budgets can be seen as the result of a context-dependent trade-off, reflecting environmental (e.g. climatic conditions [2], food availability [3], predation pressure [4]) demographic (e.g. group-size [5]) and physiological (e.g. lactation [6,7], pregnancy [8]) constraints. Moreover, individual characteristics such as sex [9,10], age [11] and dominance rank in group-living species [12] may dictate what activities require more time investment.

One of the core activities in the time budgets of many social animals is ‘allo-grooming' (hereafter: ‘grooming'), a prosocial behaviour found across multiple taxa [13–16] and extensively studied in non-human primates [17–20]. Although grooming is thought to have evolved primarily for its hygienic function [17,21], it plays a pivotal role in forming and maintaining social bonds [19,22,23] which, in turn, are linked to ultimate fitness benefits such as longer lifespans [24–26] and increased infant survival [27,28]. Grooming also functions as a tradeable commodity, *sensu* ‘biological markets' [29], given in exchange for coalition support [30,31], tolerance [32,33], protection [34], infant handling [35,36] or grooming itself [18,37]. Finally, grooming has physiological benefits and has been linked to reduced hypothalamic–pituitary–adrenal-axis activity in several primate species [38–43], which could positively affect longevity [44]. Accurate quantification of grooming is thus crucial for our understanding of its role in the above contexts.

To date, grooming data have been collected through direct behavioural observations which are typically restricted to one or a few individuals at a time and limited by both environmental (e.g. the habitat the animal lives in) and species-specific (e.g. nocturnal versus diurnal) variables [45,46]. Traditional behavioural observation methods, i.e. focal and scan sampling [47], allow researchers to approximate activity budgets by calculating rates of behaviour. This generally leads to questions about grooming being addressed in a correlative manner: e.g. are different social contexts [18,48], social partners [49,50] or physiological states [39,41,51] associated with higher or lower rates of grooming? Unless grooming data are collected in detail, usually as part of an experimental design which requires considerable observer efforts (e.g. [16,42,52]), investigating directional or dynamic relations between grooming and variables of interest is generally precluded.

The advances in animal-mounted tracking devices have allowed researchers to gain insights into animal movement and behaviour that would have been impossible to record through direct observations [45,53]. Tri-axial accelerometers allow identification of behaviours through their unique acceleration patterns [45]. In a first instance, accelerometers might simply provide information on whether the animal is ‘active' or ‘inactive' [54–56]. However, recent studies have identified specific behaviours [57,58], and some have used accelerometers to estimate activity budgets [59–61]. In an extensive review on the use of accelerometers in behavioural studies, Brown *et al*. [45] showed that the identified behaviours typically fall under the categories of ‘locomotion', ‘resting' and ‘feeding/foraging', but highlighted the general scarcity of measurements of social behaviours, with some exceptions, including mating [59,62], parent–offspring interactions [63], aggressive interactions [59] and territorial or courtship displays [64]. The limited use of acceleration data to estimate social behaviours is probably because such behavioural ‘events' occur less frequently and for shorter time periods than ‘state' behaviours [45].

Self-grooming/preening has been identified using accelerometers with varying levels of accuracy (0–50%) in a number of species [58,60,65–69], suggesting that identification of social grooming could also be possible. Primates dedicate substantial time to grooming [17] and thus present an ideal study system to collect accelerometer data on this behaviour. In fact, the first study to produce an acceleration ethogram for a primate included grooming [57]. This study was conducted on male chacma baboons (*Papio ursinus*) and successfully identified foraging, locomotion and resting with high precision (ability to minimize false positives/type 1 error: 88%) and recall (ability to minimize false negatives/type 2 error: 71%), but since adult males rarely or never groom one another, grooming data were infrequent and identification of grooming was less precise (greater than 60% precision and recall for receiving grooming, and approx. 20% for giving grooming). The accurate identification of grooming from accelerometers would allow this important behaviour to be included in accelerometer-derived activity budgets, in a species where grooming interactions underpin social bonds [19], with ultimate fitness consequences [24,27].

The present study aimed to identify grooming (giving and receiving) in wild chacma baboons using tri-axial acceleration data and machine learning. *Papio* are an ideal study genus as they spend between 5.7 and 18.9% of the day grooming [17]. The present study focuses on females who, unlike males, are philopatric, maintain long-term female–female bonds within the group [70], and spend larger proportions of their time grooming than males [71]. First, we used a random forest model [72] to identify behaviours from tri-axial acceleration, with a particular focus on grooming, following the ‘end-to-end' methods described in Fehlmann *et al*. [57]. Second, we applied this model to calculate activity budgets. Third, as studies of primate socioecology estimate activity budgets using traditional methods [47] to investigate how environmental [2,73], anthropogenic [74,75], reproductive [41,76,77] and demographic [78–80] factors and dominance rank [5,81] affect grooming rates, we compare accelerometery-based rates of behaviours with rates obtained from direct (focal) observations.

## 2. Material and methods

### 2.1. Study site and troop

The study was conducted on the ‘Da Gama’ troop which consisted of approximately 50 individuals, including two adult males and 19 adult females. The troop was studied in Table Mountain National Park and the neighbouring residential areas of Da Gama and Welcome Glen, in the Western Cape, South Africa (−34.15562° N, 18.39858° E) between June and November 2018. Research was permitted by local authorities (Cape Nature, permit number: CN44–59–6527; SANparks, permit number: CRC/2018–2019/008–2018/V1) and collaring (see below) approved by Swansea University's Ethics Committee (IP-1314-5).

### 2.2. Collars and acceleration data

SHOAL group in-house collars (F2HKv3) were built at Swansea University. Each collar contained a Daily Diary device [82] containing a tri-axial accelerometer (recording at 40 Hz continuously) and a GPS unit (GiPSy 5 tag, TechnoSmArt Italy; recording at 1 Hz between 08.00 and 20.00 local time). Collars were fitted to the baboons between 25 July and 2 August 2018 in collaboration with Human Wildlife Solutions (HWS). After entering food-baited cages, baboons were anaesthetized by a local certified veterinarian using Ketamine (dose adjusted for body mass) in accordance with local protocols (described by Fehlmann *et al*. [57]). Collars weighed mean 2.2% baboon body mass (range 1.2–2.6%) and were fitted with a drop-off mechanism (version CR-7, Telonics, Inc.) to reduce the need for recapture. No baboons died or sustained injury during capture and no injuries were observed from wearing the collars. Sixteen adults (*n* = 2 males, *n* = 14 females) were fitted with collars. One collar was not retrieved at the end of the study period (F1), one collar did not collect accelerometer data (F13), one collar only collected 2 days of acceleration data (F17, before any video data were collected; see electronic supplementary material, table S2) and one collar collected faulty data (F18, as confirmed by matching accelerometer to GPS to estimate ‘active' time; electronic supplementary material, figure S6). Together this resulted in a final sample size of *n* = 12 individuals (*n* = 10 females, *n* = 2 males; see electronic supplementary material, table S2 for individual acceleration data details), and a total of *n* = 680 collar days (mean ± s.d. = 53 ± 23 full days of accelerometer data; the first day of trapping and the last day of collar data were discarded for each baboon, to only use full days in the analyses, see ‘Activity budgets based on acceleration data').

### 2.3. Video collection and processing

Baboons were habituated to observer presence, which allowed for the collection of video data using hand-held video-recorders from a minimum distance of 10 m (Sony HD Handycam HDR-CX190). During a video-follow, the observer dictated date and time and narrated behaviours. In total, 29.4 h of video were recorded (mean ± s.d. = 2.3 ± 0.8 h per individual, range 1.3–3.5 h) from which baboon behaviours were extracted at time-steps of 1 s, generating a labelled dataset of 36 behaviours (see electronic supplementary material, table S3 for full ethogram and table S4 for ethogram sample sizes).

Some videos contained multiple collared individuals (particularly videos of grooming dyads), meaning that some video footage was used to label behaviours of more than one individual. During preliminary analysis, some behaviours (receiving grooming, resting, foraging) were sub-classified by posture (lying, sitting, standing). However, fewer behavioural categories have been found to improve model accuracy [83], and here, behavioural categories were collapsed into a single category (without posture) to improve the overall accuracy of the random forest model (analyses not shown).

We focused on six main ‘state' behaviours following Fehlmann *et al*. [57]: ‘giving grooming', ‘receiving grooming', ‘resting', ‘foraging', ‘walking' and ‘running'. We focused our analyses on these state behaviours as they are generally mutually exclusive [84] and represented the majority of the baboons' activity (94.4% of the video data). The other 30 behaviours (electronic supplementary material, table S4), which represented 5.6% (1.4 h) of the video data, were not included in any analyses (as is common, see e.g. [57,58,83,85]). Of these, 1.2% were rare behaviours (e.g. mating, aggressive interactions) and 4.4% were instances where ‘event' behaviours occurred during state behaviours (e.g. body-shakes, self-scratching, lip-smacking, see electronic supplementary material, table S3) and were removed to obtain a ‘pure' behavioural dataset. If the baboon was shifting from one behaviour to another (e.g. from sitting to walking), the adjustment period (typically less than 2 s) was assigned to whichever state behaviour most closely matched the transitionary behaviour. This resulted in 83 243 s, or 23.1 h (on average 3.9 ± 2.5 h per behaviour; and 1.9 ± 0.7 h per baboon; table 1) of video data for use in the random forest model analysis. We also extracted the number of independent events (table 1) where a new event was classified as a change in the main activity (e.g. transition from receiving grooming to giving grooming). In the calculation of independent events, additional behaviours (e.g. self-scratching, adjusting body position, lip-smacking) and changes in posture (lying, sitting, standing) were included in the same event to maintain a conservative estimate of number of events (e.g. giving grooming interrupted by self-scratching would still constitute one event).

**Table 1.** Sample sizes for random forest model training and validation. Number of seconds and independent events per behaviour recorded for each baboon (*n* = 12). For details on behaviour labelling see main text. S, number of seconds; E, number of independent events; T.S., number of training seconds; V.S., number of validation seconds.

baboon ID	resting		giving grooming		receiving grooming		foraging		walking		running		T.S.	V.S.
	S	E	S	E	S	E	S	E	S	E	S	E	S	S
M1	2666	51	1268	6	336	2	3349	67	505	83	160	34	5749	2535
M2	563	14	364	6	1151	2	377	4	267	25	28	6	1932	818
F2	1146	22	734	3	1307	6	1357	33	383	58	70	20	3527	1470
F4	1642	32	2352	17	3054	15	2535	98	867	110	45	8	7366	3129
F5	776	36	4238	23	1094	9	2769	89	840	107	0	0	6760	2957
F6	1120	24	3079	22	1359	15	1557	45	836	71	47	14	5605	2393
F7	1224	11	292	7	0	0	2416	40	415	53	16	4	3082	1281
F9	831	27	1578	15	1690	8	429	17	226	40	33	6	3335	1452
F10	707	39	3257	30	2868	27	3160	96	1009	130	77	22	7771	3307
F14	1322	25	1870	17	1241	17	811	27	262	34	18	5	3870	1654
F15	287	20	2677	19	1007	10	2062	78	961	103	9	3	4869	2134
F19	1900	40	319	5	436	10	2983	74	602	87	7	2	4388	1859
total	14 184	341	22 028	170	15 543	121	23 805	668	7173	901	510	124	58 254	24 989

### 2.4. Acceleration data preparation

The analysis of accelerometer data detailed below closely follows the workflow and code provided by Fehlmann *et al*. [57] and used Daily Diary Multi Trace (DDMT; http://www.wildbytetechnologies.com) software. Before baboons were fitted with the collars, sensors were calibrated at the field site to create offsets in DDMT, providing the time reference used to match video to accelerometer data. The position of the daily diary in the collar was specified to correct the position of acceleration channels (X = surge, Y = sway, Z = heave) relative to the ground. Datasets containing the labelled behaviours with associated timestamp were imported into DDMT as ‘bookmarks'. Timestamps were verified visually to ensure the DDMT timestamp matched the video timestamp. Accelerometer data and associated behaviours were exported out of DDMT using Bookmark Multisession.

### 2.5. Computing variables from acceleration data

All analyses (computation of variables, random forest models, comparison of models and calculation of activity budgets) were conducted in R studio (version 3.6.1). Tri-axial acceleration allows the identification of behaviours through deriving information about the posture of the animal (static acceleration) and the movement of the animal (dynamic acceleration). Calculations combining the signal along the three axes can provide further metrics that can be used to differentiate behaviours from one another. To match the labelled behaviours (1 Hz) to the acceleration signal (40 Hz), the mean values were calculated for 16 acceleration variables per second. Acceleration variables were computed using the methods described in Fehlmann *et al*. [57], excluding the variables that were found to have low predictive power. This resulted in the following 16 variables being included in the model: (1–3) tri-axial static acceleration (X, Y, Z), (4–5) pitch and roll, (6) vectorial dynamic body-acceleration (VeDBA), (7) smoothed VeDBA (VeDBAs), (8–10) tri-axial partial dynamic body acceleration (PDBA) and (11–16) tri-axial power spectrum density (PSD) for the first and second associated maximum frequencies. See Fehlmann *et al*. [57] for a comprehensive description of these variables and associated R script.

### 2.6. Random forest model fitting

Random forest models have been employed for many accelerometer-derived behaviour identification studies and have been found to outperform other machine learning approaches [85–88]. To run the random forest models, we used the R package ‘random forest' [89]. Random forests are a machine learning method based on building classification trees [72]. It operates using two ‘layers of randomness' by first using a random subset of the data each time a tree is grown, and second by using a random subset of variables (here the 16 variables computing from tri-axial acceleration) for each classification step [90]. Each classification tree contains a set of hierarchical decision rules which aims to split the data into subsets which represent a given behaviour. To achieve ‘purity’ in the subset (pure = a subset which only contains one behaviour), decision rules aim to maximize the impurity reduction at each split. Some variables may contribute more to the decrease in impurity at each split than others, and this can be represented by the Gini index: the difference between the impurity at a split and the sum of the weighted impurity of the two splits that follow, averaged across all trees [91]. The Gini index can be used to rank the variables used to build the random forest in order of importance. Following Fehlmann *et al*. [57], we ran the random forest model with 500 trees and left the parameters at default. To confirm no further iterations (i.e. trees) were required for the model to stabilize (i.e. obtains the best classification results), we ran a *post hoc* test which revealed that error rates level out after 100 iterations (electronic supplementary material, figure S3).

The labelled dataset from all 12 baboons was divided at random into a 70% (58 254 s; 16.2 h) training set and a 30% (24 989 s; 6.9 h) validation set (to test the precision and recall of the random forest model prediction) allowing for a ‘supervised algorithm' approach [57,66,92]. Behavioural classes and individuals were equally represented in the training and validation dataset (variation within 1%; see electronic supplementary material).

### 2.7. Model validation

Using the random forest model generated with the training set, we predicted the behaviours from the validation set by running 500 trees [57] where the most frequently predicted behaviour across 500 trees is presented as the final prediction. To assess recall and precision, we compared the output from the predicted behaviours with the observed behaviour in a confusion matrix (table 2) using the calculations below.

 Precision=TP(TP+FP)  and Recall=TP(TP+FN),

where TP is true positive; TN, true negative; FP, false positive; FN, false negative.

**Table 2.** Confusion matrix for the random forest model. Comparison of the predicted behaviour (rows) and observed behaviour (columns), based on labelled dataset from videos. Values in italics represent the true positives (TP). Instances where the behaviour was incorrectly classified by the model (false positives: FP) are in rows, instances where the behaviour was missed by the model (false negatives: FN) are in columns.

behaviour	resting	receiving grooming	giving grooming	foraging	walking	running	total predicted
resting	*3243*	416	246	180	9	9	4094
receiving grooming	182	*3243*	158	14	0	0	3953
giving grooming	438	429	*5816*	500	0	0	7183
foraging	331	122	435	*6151*	411	3	7453
walking	18	3	4	266	*1701*	17	2009
running	0	0	0	4	18	*122*	144
total observed	4212	4569	6659	7115	2139	142	

### 2.8. Activity budgets based on acceleration data

To obtain activity budgets for each baboon across their respective collar periods, the model output from all baboons was applied to the entire accelerometer dataset (16 319 h; 680 days, *n* = 12 baboons) to estimate the total number of seconds engaged in each behaviour. To allow for the comparison of activity budgets from collars with those obtained from direct focal observations, a subset of the accelerometer data was used, corresponding to the time window covering direct observation hours (between 07.00 and 17.00 local time).

Of the total 58 747 636 s (16 319 h; 680 days) of accelerometer data, 316 654 s (88 h, 0.54% of the total dataset) could not be classified as one of the six behaviours (mean ± s.d.: 28 786 ± 76 359 s, 8 ± 21 h per individual (*n* = 11); median: 3176 s, 0.9 h). One individual had no non-classified behaviours. As these points could not be definitely assigned to any behaviour, they were removed from the daily budget calculations. Further investigation into the characteristics of non-classified acceleration datapoints is provided in the electronic supplementary material (‘Non-classified behaviours'; electronic supplementary material, figure S5). One individual (F18) was excluded from further analysis and removed from the random forest model analysis (see electronic supplementary material, figure S6) because we observed a discrepancy between accelerometer and GPS-identified activity.

### 2.9. Activity budgets based on focal data

Focal observations [47] were conducted for all collared individuals (*n* = 16, of which *n* = 12 have acceleration data) between August and November 2018 (two observers: CC, AMB) and included both an instantaneous and a continuous component. Instantaneous data were collected on activity (grooming, resting, foraging, walking, running or engaged in other social behaviour) every minute for 30 min, resulting in 31 records per focal observation [93]. All social interactions (including giving and receiving grooming) were recorded in detail in the continuous part of the focal observation to the nearest second. If the grooming interaction was still ongoing by the end of the 30 min focal period, the focal observation was continued until the end of the grooming bout (following [94]). For the instantaneous data, rates of behaviour were calculated by dividing the number of scans engaged in each behaviour by the total number of scans. For the continous grooming data, rates were obtained by dividing the total time (seconds) engaged in giving or receiving grooming by the total observation time. While grooming rates are typically calculated based on adult grooming interactions [18,19], for the purpose of comparing focal with accelerometer-identified grooming rates (see below), all grooming interactions (including grooming with juveniles and non-collared adults) were included, as grooming partner identity is not distinguishable in accelerometer-identified grooming. Focal observations were carried out within five time-blocks (07.00–09.00, 09.00–11.00, 11.00–13.00, 13.00–15.00, 15.00–17.00; electronic supplementary material, figure S1) and individuals were observed in a randomized order across time-blocks.

Focal data were collected up to the collar drop-off date (16 October 2018) and only focal observations of more than 3 min in length were used. In total, *n* = 323 focal follows were conducted (mean ± s.d. = 27 ± 4 per individual), the equivalent of 154 h (mean ± s.d. = 13 ± 2 h per collared individual, *n* = 12). First, we used a Spearman's correlation to establish whether rates calculated from the full focal dataset were correlated with those obtained when only using the focals collected while the collars were recording data (i.e. a ‘true time match'), with the focal data window adjusted for each baboon's collar duration (*n* = 208 focals, mean ± s.d. = 17 ± 9 per individual; *n* = 97 h, mean ± s.d. = 8 ± 4 h per individual). As the correlations were strong (*ρ* range: 0.77–0.92) and highly significant (*p* ≤ 0.005 for all behaviours; electronic supplementary material, figure S2), all focal data were included to maximize the amount of data used in the analysis. Second, to test if relative rates obtained from the collars (see ‘Activity budgets based on acceleration data') were positively correlated with the rates from focal observations, we used Spearman's correlations. Further, to test whether rates were consistently higher or lower for different behaviours when using focal or collar data (which would indicate a method-based bias), we used Wilcoxon signed-rank tests.

## 3. Results

### 3.1. Acceleration ethogram

**Figure 1.**
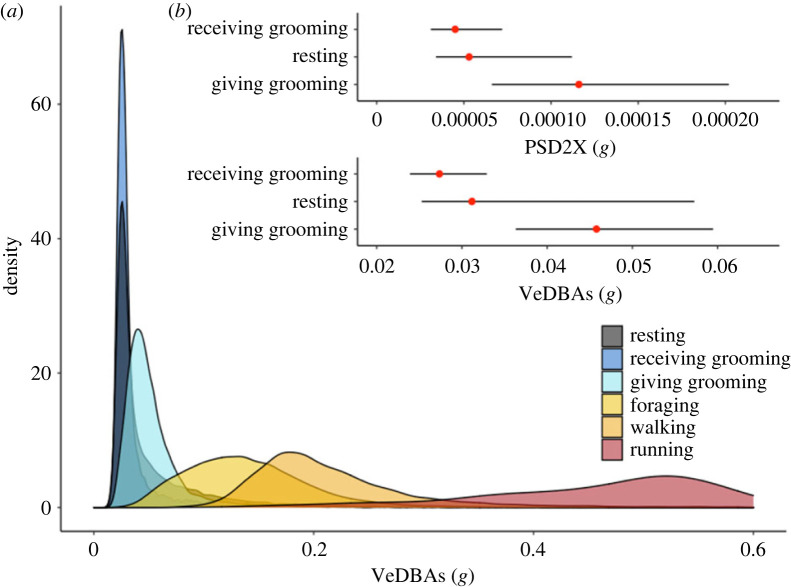
Acceleration ethogram. (*a*) Density plots of smoothed VeDBA (VedBAs; most important predictor variable for random forest model) for the six state behaviours. Note that the ‘running' density plot extends beyond 0.6*g* but was cropped for better visualization of the other behaviours. (*b*) Interquartile ranges for PSD2X and VeDBAs with the median (red dot) for the three stationary behaviours: resting, receiving grooming and giving grooming. Median VeDBAs for the three behaviours fall within the interquartile ranges of another stationary behaviour. Conversely, median PSD2X for giving grooming falls outside the interquartile ranges of resting and receiving grooming (electronic supplementary material, table S7).

**Figure 2.**
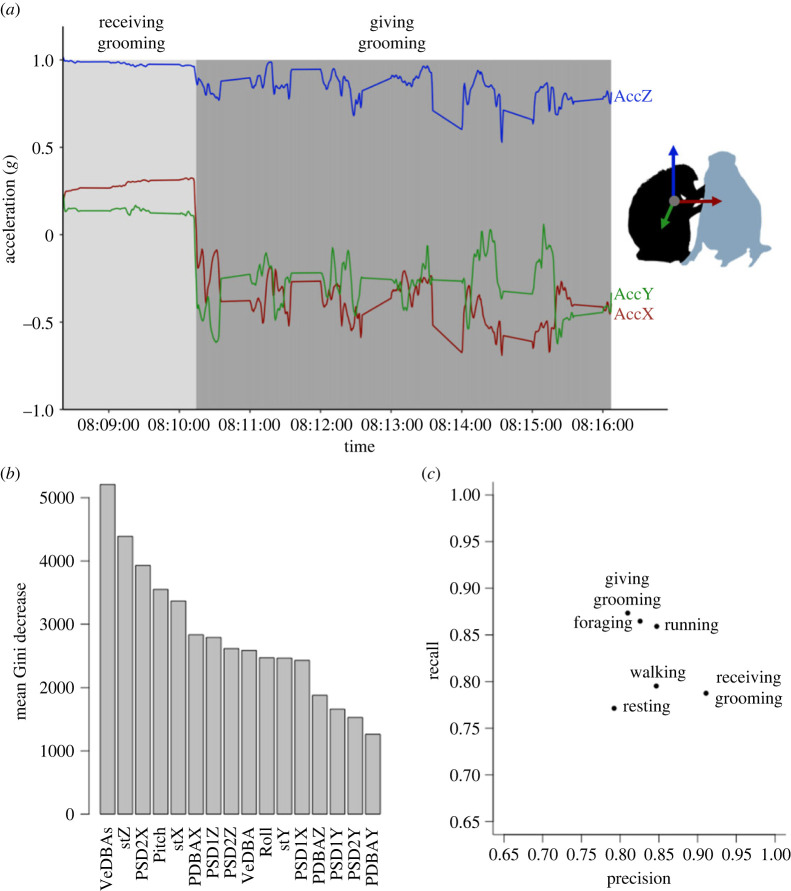
Random forest model results. (*a*) Example of labelled tri-axial acceleration data from a female baboon switching from receiving grooming to giving grooming (AccX = surge (red), AccY = sway (green), AccZ = heave (blue)) over an 8 min period. (*b*) Mean Gini decrease, ranking the variables in order of importance for identification of baboon behaviours in the random forest model. (*c*) Precision and recall for six identified behaviours from the random forest model.

Smoothed VeDBA (VeDBAs) was the most important variable for distinguishing among behaviours (figure 2*b*). VeDBAs during running (median [first and third quartile]: 0.85*g* [0.62*g*–1.09g]) showed no overlap with any other behaviour, and foraging and travelling had medians that fell outside the interquartile ranges of all other behaviours (figure 1*a*; electronic supplementary material, table S7). Conversely, the three ‘inactive' behaviours (giving grooming, receiving grooming and resting) showed substantial overlap in VeDBAs ranges (electronic supplementary material, table S7; figure 1*b*). Median VeDBAs for resting (0.031 g) was slightly higher than for receiving grooming (0.027 g) (electronic supplementary material, table S7), which probably led to the overestimation of receiving grooming during the night (see ‘Activity budgets').

Static acceleration along the heave and surge axes (both provide information on posture) was also important, with stZ and stX ranked second and fifth, and pitch (forward/backward rotation) ranked fourth (figure 2*b*). The interquartile ranges for static acceleration channels overlapped for all behaviours, but the interquartile range for receiving grooming was consistently the largest followed by resting (see electronic supplementary material, table S7 and figure S4 for distribution of mean stZ), suggesting that a large range of postures is adopted during these behaviours.

Three of the power spectrum densities (PSDs), i.e. PSD2X, PSD1Z, PSD2Z were in the top 10 most important variables. Notably, PSD2X was important for identifying giving grooming with a median that did not fall within the interquartile ranges of other behaviours (median [first and third quartile]: 0.0001*g* [0.00007*g*–0.0002*g*]; figure 1*b*). This suggests that giving grooming occurs on a regular low-amplitude frequency (with lower values than the aforementioned ‘active' behaviours but higher than the two other ‘inactive behaviours', viz., resting and receiving grooming).

### 3.2. Model performance

The model reached a precision of mean ± s.d. = 83.8 ± 0.4% and a recall of mean ± s.d. = 82.5 ± 0.5% (electronic supplementary material, table S6). Receiving grooming had 91% precision and 79% recall, while giving grooming had 81% precision and 87% recall (figure 2*c*). Resting had the lowest precision (79%) and recall (77%) and was mostly confused with giving or receiving grooming (table 2; figure 2*c*). Walking, foraging and running had high precision and recall (greater than 80%; figure 2*c*). The slightly lower recall for walking compared with other active behaviours was primarily due to instances misclassified as foraging (table 2), probably caused by the intermittent nature of walking and foraging.

### 3.3. Activity budgets

Activity budgets were calculated by applying the random forest model to the dataset (24 h d^−1^, total collar days = 680; electronic supplementary material, table S8). Baboons spent on average (mean ± s.d.) 21.4 ± 9.0% of their time resting, 18.8 ± 6.3% giving and 30.0 ± 8.1% receiving grooming (*n* = 12; electronic supplementary material, table S8). When restricting the collar data to direct observation hours (07.00–17.00) the baboons spent 19.0 ± 9.6% of their time resting, 18.7 ± 6.6% giving grooming and 15.2 ± 6.5% receiving grooming (*n* = 12; electronic supplementary material, table S9). See electronic supplementary material, tables S8 and S9 for active behaviours (foraging, walking and running).

Based on the results of the accelerometer-identified activity budgets, which suggested receiving grooming may be confused with resting (particularly during the night), we calculated whether VeDBAs (the most important predictor variable; figure 2*b*) associated with resting overlapped more with receiving grooming during the night than during the day, which was the case (see ‘Night versus Day: Resting versus Receiving grooming VeDBAs' in electronic supplementary material for details).

### 3.4. Comparing acceleration-based rates of behaviours with focal rates

**Figure 3.**
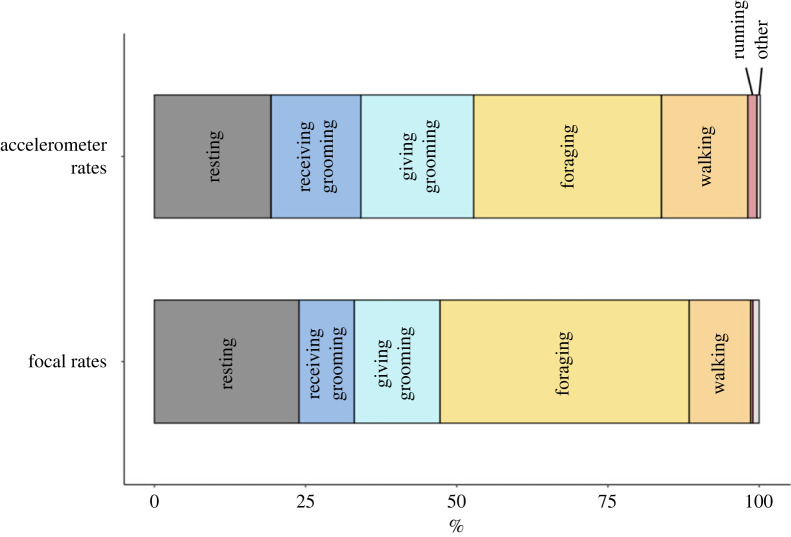
Overall accelerometer-identified and focal activity rates for six main behaviours. Mean rates across *n* = 12 baboons estimated by accelerometer data using the random forest model (top bar) and normalized focal rates calculated from direct observations (lower bar, see electronic supplementary material, table S13).

**Figure 4.**
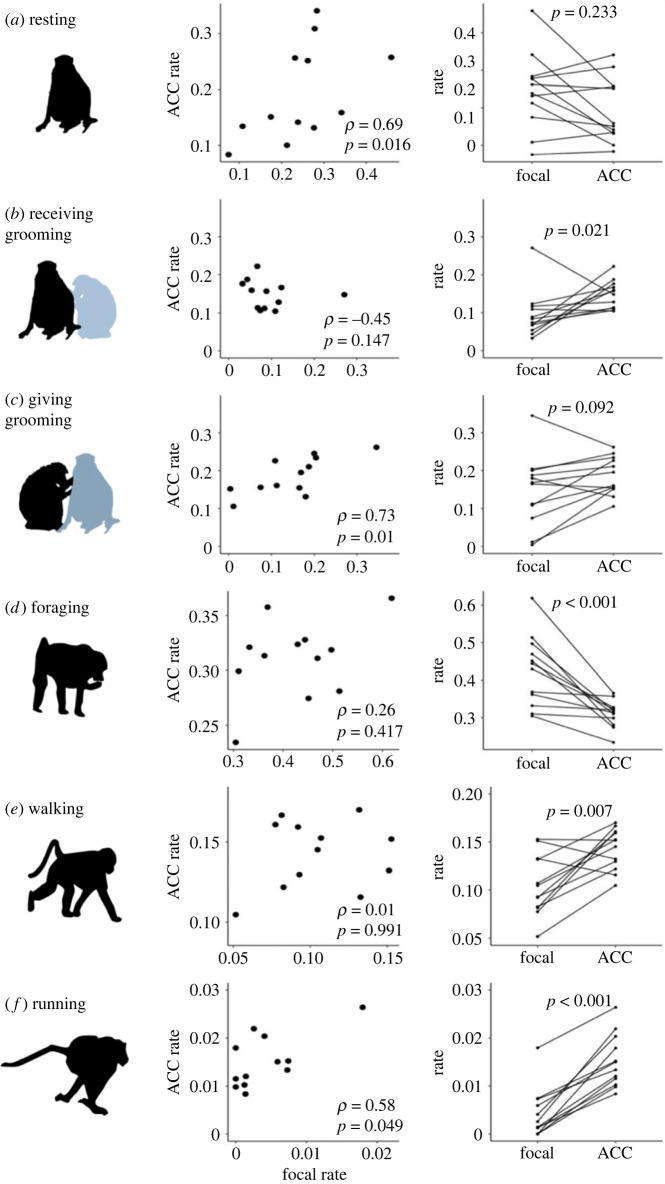
Individual accelerometer-identified and focal rates. Rates of behaviours calculated from focal and acceleration (ACC) data (*n* = 12 baboons) showing the Spearman's correlation (left) and Wilcoxon signed-rank test comparison (right) between rates obtained with the two methods: (*a*) resting, (*b*) receiving grooming, (*c*) giving grooming, (*d*) foraging, (*e*) walking and (*f*) running. Note that axes scales differ between behaviours.

Overall behavioural budgets (during observation hours of 07.00–17.00) calculated using focal and accelerometer data (based on random forest models) revealed comparable activity budgets (figure 3; electronic supplementary material, table S13). Behavioural rates obtained from accelerometer-identified budgets were significantly correlated with focal rates for giving grooming (*ρ* = 0.73, *p* = 0.010, *n* = 12; figure 4*c*), but not for receiving grooming (*ρ* = −0.45, *p* = 0.147, *n* = 12; figure 4*b*). Rates from accelerometer and focal data were significantly correlated for resting (*ρ* = 0.69; *p* = 0.016, *n* = 12; figure 4*a*) and running (*ρ* = 0.58, *p* = 0.049, *n* = 12; figure 4*f*) but not for foraging (*ρ* = 0.26; *p* = 0.417, *n* = 12; figure 4*d*) and walking (*ρ* = 0.01, *p* = 0.991, *n* = 12; figure 4*e*). Focal sampling resulted in lower rates of receiving grooming (Wilcoxon signed-rank test: *Z* = −2.31, *p* = 0.021, *n* = 12; figure 4*b*) but not significantly different rates of giving grooming (Wilcoxon signed-rank test: *Z* = −1.68, *p* = 0.092, *n* = 12; figure 4*c*) compared with accelerometer data. Focal sampling showed higher rates of foraging (Wilcoxon signed-rank test: *Z* = −3.49, *p* < 0.001, *n* = 12; figure 4*d*), but lower rates of walking (Wilcoxon signed-rank test: *Z* = −2.70, *p* = 0.007, *n* = 12; figure 4*e*) and running (Wilcoxon signed-rank test: *Z* = −3.49, *p* < 0.001, *n* = 12; figure 4*f*). There was no significant difference in resting rates between the two methods (Wilcoxon signed-rank test: *Z* = −1.19, *p* = 0.233, *n* = 12; figure 4*a*).

## 4. Discussion

This study aimed to quantify grooming from accelerometer data using machine learning. We first used random forest models to identify receiving and giving grooming (and other behaviours) from accelerometer data collected from *n* = 12 collared wild chacma baboons. Second, we applied the random forest model to calculate activity (grooming) budgets for each individual. Third, we compared rates of grooming obtained from focal data (direct observation) with rates obtained from accelerometer data (using the random forest model). Below, we discuss each objective and associated findings in turn. We also discuss the implications of this methodological advance for the study of social grooming and future avenues for its application.

This study is the first to identify grooming with high precision and recall for both actors (precision 81% and recall 87%) and receivers (precision 91% and recall 79%) using tri-axial accelerometer data. Compared with previous work on male baboons only [57], the focus on females (who devote high proportions of the day to grooming [71]) and the larger sample sizes for both grooming behaviours (± 6 h versus ± 1.5 min for giving grooming; ± 4.5 h versus ± 4 min for receiving grooming in the current versus previous dataset, respectively) probably explain this improved precision and recall (e.g. [58]). This demonstrates that grooming behaviour, if performed frequently and when targeted during video follows by the researchers, can be successfully identified and included into activity budget as social behaviour alongside other state behaviours [45]. Because machine learning is a ‘black box' in terms of its internal decision rules [92], it is important to consider what biomechanical features distinguish grooming from other stationary behaviours in acceleration signals [95]. Below we describe the findings for giving and receiving grooming in turn.

Acceleration profiles for giving grooming show that sufficient movement takes place to produce a distinctive cyclic pattern, with a median PSD2 along the *X*-axis (surge) which falls outside the interquartile ranges of any other behaviours (electronic supplementary material, table S7, figure 1*b*). This surge (back-and-forth) motion makes sense when considering the typical grooming rhythm, in which the actor repeatedly moves their hands forwards and across the recipient in front of them. Power spectra are typically used for identifying locomotion which produces repeated oscillations [57,69,96,97], but self-grooming in domestic cats (*Felis catus*) is also associated with differently paced cyclic patterns along the surge axis [65]. In a study on dingoes (*Canis dingo*), ‘self-grooming’ was classified as a ‘medium' activity class (repetitive head movement) associated with higher overall dynamic body activity (ODBA) compared with resting behaviours [85]. Moreover, giving grooming takes place in a relatively consistent posture (sitting), which narrows the range of static acceleration associated with this behaviour (e.g. electronic supplementary material, figure S4) compared with studies of self-grooming where postures may vary depending on the body part being cleaned [65,68].

Taken together, the present and previous studies suggest that the act of giving grooming, while stationary, can still produce a distinct acceleration pattern that is discernible from other stationary behaviours (i.e. resting, receiving grooming). Our study also highlights the value of performing further waveform analyses to obtain descriptive statistics of how signal varies across time (e.g. to detect repetitive patterns), rather than relying on measures of general body activity. For instance, previous work on captive rhesus macaques using omni-directional accelerometers (which provide a general indicator of ‘physical activity') successfully differentiated ‘active' from ‘inactive' behaviours [56], but these were unaffected by arm and neck movements which would be important for identifying grooming. This corresponds to our findings: VeDBAs (also a general measure of activity based on the dynamic acceleration across the three axes) overlaps between the three stationary behaviours (resting, receiving and giving grooming; electronic supplementary material, table S7, figure 1*b*). By contrast, the relatively small but repetitive movements during giving grooming were discernible in the PSDs (figure 1*b*).

For receiving grooming, the acceleration profile presents a challenge due to its resemblance to resting (the difference between sitting versus sitting while being groomed is inevitably subtle). Previous studies testing the use of tri-axial accelerometry to differentiate between non-active or slowly executed behaviours suggest that there are limits to what fine-scale changes in movement can be detected using accelerometers alone [98,99]. As both resting and receiving grooming are executed in similar body positions (e.g. sitting, lying), static acceleration—which informs posture—would not be sufficient to distinguish between these behaviours (e.g. see electronic supplementary material, figure S4 for stZ distribution). Nevertheless, the random forest model shows relatively high precision and recall for both behaviours (greater than 77%). Dynamic acceleration and its derivatives must thus pick up on very small changes in movement. While being groomed may be expected to be associated with slightly more body movements due to the manipulation of fur by another baboon, the median VeDBAs is, in fact, lower for receiving grooming than for resting (electronic supplementary material, table S7; figure 1*b*).

There are several reasons why resting may generate more overall body movement than receiving grooming. First, based on the labelled dataset, daytime resting is a behavioural state that is relatively brief compared with receiving grooming (electronic supplementary material, table S5; resting bouts were on average four times shorter than receiving grooming bouts). Thus, resting does not necessarily reflect uninterrupted periods of relaxation (which would presumably be associated with very low VeDBAs), but rather takes place as a relatively brief pause between activities. Consequently, when taking a moving average across a 3 s window (as was done to obtain VeDBAs), the calculation will take into account a second of the behaviour that precedes and follows resting. Quick transitions can result in misclassifications of behaviours [100], and, considering that transitions have distinct acceleration signatures [101], it is possible that more noise is introduced into the resting signal due to its intermittent nature compared with receiving grooming, which is longer in duration. Second, from a biological perspective, resting during the daytime (when video footage was collected), may be a more active behaviour than the name suggests. While standing (resting quadrupedally) and secondary behaviours such as self-scratching and body shakes were removed from the dataset to create a ‘purer' resting category, baboons still move their body during resting when scanning the environment (e.g. vigilance [102]) or as they prepare to start moving [103]. Conversely, when being groomed, baboons typically stay still, as would be expected when considering the tension-reducing effect of being groomed, which is reflected in lower rates of behavioural indices of stress (e.g. yawning, scratching, body-shaking, auto-grooming [104]). Furthermore, staying motionless makes the removal of ectoparasites during grooming more effective [104].

The above notwithstanding, receiving grooming and resting are similar from an accelerometry perspective with a difference in VeDBAs of less than 0.1*g* (figure 1*b*). Essentially, this reduces the distinction to: was the baboon ‘still' or ‘very still'? Based on the results from the random forest model, ‘still' corresponds to ‘resting’ and ‘very still' corresponds to ‘receiving grooming' in a relatively reliable way within the training data (approx. 23 h), but the assumption may not hold across all contexts for the full study period (16, 319 h), which should be borne in mind when interpreting receiving grooming budgets (see below). Future studies, or further exploration of this dataset, could investigate ways to distinguish receiving grooming and resting with more certainty. Using GPS-identified dyads may help, i.e. if two baboons are spatially close and are classified as giving and receiving grooming, respectively, this may further help confirm receiving grooming. However, baboons often groom in small sub-groups [105], and it is not uncommon to see several females grooming and resting in proximity without necessarily grooming one another (C Christensen, AM Bracken 2018, personal observation). Alternatively, accelerometers have been deployed on different body parts to target or tease apart similar (from an accelerometry point of view) behaviours [45,106]. For instance, mandible- and head-mounted accelerometers deployed on seals have been used to distinguish between feeding and vocalizing (*Leptonychotes weddellii* [107]) and between resting and being alert (*Halochoerus grypus* [63]), respectively. Arm-mounted accelerometers have been used in captive baboons [108] and wrist-mounted accelerometers are currently being tested in wild olive baboons [109]. Self-directed behaviours (e.g. scratching) decline while receiving grooming [104] and can increase during resting [110,111], thus wrist-mounted accelerometers may add a layer of information that could help distinguish between the two behaviours. Finally, collar-mounted cameras deployed on primates have recorded grooming [112], but this data collection method is costly in terms of energy and on-board memory (B Walton 2022, personal communication). In conclusion, resting and receiving grooming fall into a category of stationary behaviours that present challenges for identification using tri-axial acceleration signals alone [98]. Collar-mounted tri-axial accelerometers do allow estimating these behaviours to an extent, but continued efforts to improve detection will allow leveraging the benefits of continuous grooming data with more certainty.

The ability to quantify grooming continuously (as made possible by using collars) opens a wealth of potential questions for investigation, for instance in the fields of socio-endocrinology [20], socioecology [2,113], biological markets [18,29] and grooming social networks [114]. Rather than correlating grooming rates to concurrent physiological, ecological or social conditions, continuous grooming data from collars allow tracking the dynamic nature of the decision-making process during grooming. For example, simultaneous grooming activity across a social network could shed light on how much time and when individuals invest in grooming relative to the changing availability of grooming partners. Collars also allow measuring grooming at times when direct observation is precluded, such as at hard-to-reach sleep sites or at night [115–117]. Physiological correlates of grooming, which are often monitored non-invasively in the wild (e.g. urine and faeces [118]), can be studied by time-matching hormone measures to grooming data retrospectively; addressing both how grooming is affected by physiology and vice versa, e.g. in the context of social buffering [119] or of the sociality–health–fitness relationship more broadly [120]. Finally, while the grooming literature is primate-skewed, many other group-living animals perform allo-grooming [13,16,23,121] or allo-preening [14] and use it as a tradeable commodity [16] and/or in social bond formation [23]. As tracking devices have been deployed successfully in some of these systems (e.g. *Suricata suricatta* [95], *Desmondus rotundus* [122], *Equus caballus* [123]), grooming identification from acceleration data could be applied more widely, particularly if the postures adopted during grooming are relatively consistent (e.g. as was the case for giving grooming in this study).

Our second objective—calculating activity budgets based on random forest models—revealed an important aspect of the evaluation of random forest model performance. The accelerometer-identified activity budgets across 24 h suggest that overall baboons spent on average 30% of time engaged in receiving grooming, 19% giving grooming and 21% resting. When restricting the time window to direct observation times (07.00–17.00), baboons spent on average 15% receiving grooming, 19% giving grooming and 18% resting. The steep increase in receiving grooming (±15%) when including night hours, compared with a modest increase in resting (±2%), strongly suggests that resting during the night is misclassified as receiving grooming (though some of the receiving grooming is probably ‘true', as baboons are known to groom at the sleep site at night; [117]). As discussed above, there is considerable overlap between accelerometer variables between resting and receiving grooming, but receiving grooming has the lower median VeDBAs (electronic supplementary material, table S7; figure 1*b*). This raises the question whether receiving grooming is more prone to confusion with resting during the night compared with the day, which may be the case if resting during the night is more still (due to sleeping) compared with resting during the day (where brief resting is more typical; electronic supplementary material, table S5). Indeed, other studies treat resting and sleeping as separate behaviours due to the difference in energetic demands [124,125] and a recent study investigating baboon sleep patterns using accelerometers likewise distinguished between ‘sleep' and ‘resting wakefulness' [117].

The median/range VeDBAs calculations for resting during day versus night in the present study are in line with this, showing more overlap in VeDBAs between resting and receiving grooming at night (electronic supplementary material, figure S7). As a consequence, when the random forest model is trained using a biased testing dataset (e.g. daytime resting but no night-time sleeping), it can appear to function well in the first phase (e.g. when precision and recall are calculated using the same biased dataset) but, once applied to a new independent dataset, make predictions that are comparable to random guesses [126]. When considering direct observation hours only (when videos were collected), however, the activity budgets are biologically more plausible. Calculating activity budgets could serve as a ‘quality control' step for random forest model performance beyond the commonly used metrics (e.g. precision, recall).

As a third and final objective, this study offered a unique opportunity to compare acceleration-based activity budgets with direct focal observations [47]. Usually, this is not possible because the primary motivation for using collars is to reconstruct activity budgets of animals that are not readily observable in their natural habitat (e.g. [45,61]). Focal data may over- or under-estimate behaviours depending on the visibility of the behaviour or observer bias to start (or stop) focal follows during certain behaviours (e.g. stationary) over others (e.g. running) [93]. Moreover, focal data can be collected using both continuous (here: giving and receiving grooming) or instantaneous (all other behaviours) sampling methods [47,93], with the latter method being prone to underestimation of rare behaviours [93] which accelerometer data, collected at a 1 s resolution, can reliably record.

The relative breakdown of behaviours was comparable between the two methods, with baboons spending most of the day foraging, followed by resting, giving grooming, receiving grooming, walking and running (electronic supplementary material, table S13; figure 3). We found positive correlations between accelerometer and focal rates for giving grooming and resting but not receiving grooming (figure 4). Moreover, we found that receiving grooming rates were significantly lower using direct observation (figure 4*b*), while giving grooming (figure 4*c*) and resting (figure 4*a*) were comparable between the two methods. These findings suggest focal data are returning both different and relatively lower individual rates for receiving grooming, but not the other two stationary behaviours. The lack of correlation might be due to the relatively small range in individual rates of receiving grooming (between 3% and 12%, when ignoring the outlier (M2) visible in figure 4*b*), compared with giving grooming and resting ranges (0–35% and 8–46%, respectively) in the focal data, which could make it harder to detect individual differences in receiving grooming and thus correlate to accelerometer-identified rates. Receiving grooming was also the least frequent of the three stationary behaviours, both in focal and collar identified rates (electronic supplementary material, table S13; figure 3), and thus could be subject to lower estimations in focal data despite being easy to observe. Finally, accelerometer-identified receiving grooming, while more distinct from resting during the day (electronic supplementary material, figure S7), could still be confused with daytime resting if VeDBAs is low (figure 1). This would inflate the ‘false positive' receiving grooming rate returned by the accelerometers relative to focal data, causing the significantly higher rates identified in accelerometer data (figure 4*b*).

Foraging rates were significantly higher in focal data, while travelling and running were both lower using focal data. Foraging and walking occur intermittently, and it is possible that the amount of walking between foraging patches is estimated to be lower through instantaneous sampling (i.e. even if a few steps are taken between foraging patches, the behaviour would still be labelled ‘foraging' during scan sampling). Moreover, active behaviours such as walking and running are probably underestimated as the focal individual is more easily lost ([93]; C Christensen, A Bracken 2018, personal observation). Finally, sample collection (faeces and urine) as part of a larger study [118,127] often resulted in termination of observations if the focal animal moved off, which could result in less walking/running being recorded. Taken together, these findings suggest that while overall activity budgets are comparable (figure 3), individual receiving grooming rates are lower and not correlated with accelerometer-identified rates.

Overall, this study presents a step towards the quantification of social grooming in unprecedented detail, but its applicability largely depends on the specific research aim(s) and the feasibility of using collars. Deploying collars requires ethical [128], logistical (e.g. deployment, collar failure) and financial considerations [129], whereas traditional observations can be conducted with minimal interference and at low cost. Moreover, depending on the objectives of the study, direct observations may be preferable if the aim is to reconstruct entire social networks, as collar data is limited to the number of collared individuals. On the other hand, studies which aim to uncover mechanistic details about grooming stand to gain by pursuing accelerometer-identified grooming, as changes in grooming durations and frequencies can be tracked and time-matched to changes in internal (e.g. physiology) and external (e.g. environmental and social) factors.

## Data Availability

The code to run the random forest model and the video-labelled accelerometer dataset used to train and validate the model are provided as electronic supplementary material. The behavioural rates calculated from accelerometer and focal data are attached as electronic supplementary material. A document explaining the content of each electronic supplementary material is provided. The code to calculate the variables from the accelerometer data is published in Animal Biotelemetry (open access): [57]. The data are provided in electronic supplementary material [130].
